# Sustainability Literacy of Older People in Retirement Villages

**DOI:** 10.1155/2014/919054

**Published:** 2014-12-22

**Authors:** Bo Xia, Jian Zuo, Martin Skitmore, Laurie Buys, Xin Hu

**Affiliations:** ^1^School of Civil Engineering and Built Environment, Queensland University of Technology, Garden Point Campus, 2 George Street, Brisbane, QLD 4001, Australia; ^2^School of Natural and Built Environments, University of South Australia, City East Campus, North Terrace, Adelaide, SA 5001, Australia; ^3^School of Design, Queensland University of Technology, Garden Point Campus, 2 George Street, Brisbane, QLD 4001, Australia

## Abstract

With many developed countries experiencing the aging of the population, older people play a large role in contributing to environmental problems but also to environmental solutions. The purpose of this research is to understand the awareness and behavior of current older people living in retirement villages towards sustainability development. To achieve this, a sustainability literacy survey was conducted with 65 older residents of a private retirement village located 10 Km outside the Brisbane, Australia's central business district (CBD). Most of residents recognized the importance of environment protection and would like to lead a more environmentally friendly lifestyle. In addition, the majority were willing to pay higher prices for a living environment with sustainable features. The importance of positive social communications was emphasized with most residents having established good relationships with others in the village. The findings provide an important insight into consumer perspectives regarding the sustainable features that should and can be incorporated into the village planning and development.

## 1. Introduction

Sustainable development, defined as “development that meets the needs of the present without compromising the ability of future generations to meet their own needs” [1], has gained momentum in the current era and visualizes a cleaner environment, the efficient use of natural resources, and a more inclusive society with widely shared benefits of increased economic prosperity [2]. In terms of the built environment, this implies the conservation of a wide variety of resources, such as energy and water [3].

With the increasing proportion of aging population around the world, the actions of older people are likely to have a corresponding increasingly important role in the sustainable development of the community. Older people are expected to consume more energy due to both their increased numbers and their lifestyles, such as spending more time at home (requiring more electricity and water) and using their cars frequently [4, 5].

However, older people can also make major contributions to solving environmental problems [6, 7]. Older people are generally aware of resource consumption and would like their habitat and community to be more sustainable [8]. They can therefore play a positive role in engaging in sustainable activities, such as recycling, food production, renewable energy use, and using alternative transport options. Additionally, environmental volunteerism creates opportunities for social integration in later life, offering meaningful civic engagement in productive activities while providing volunteer resources to promote environmental stewardship [9].

Due to the increasingly significant potential impact of older people on both environmental problems and solutions, it is important to understand their current attitudes and behaviors concerning sustainable development, considered as one of the highest priority topics for research on aging and environmental sustainability [9]. Older people may experience reduced physical capabilities and particular ergonomic requirements (easy access, companionship, security, etc.) and generally value a combination of independence, security, friendships, and community support [10]. The realization of the sustainable agenda (e.g., sustainable buildings and environmental friendly lifecycle) needs to take into consideration these requirements. Furthermore, due to changes in financial circumstances during retirement, additional costs involved in sustainable development inevitably become a major concern [11]. A clear understanding of older peoples' perceptions of sustainable development is, therefore, necessary for devising sustainability policies and programs.

However, few studies have documented older people's attitudes and knowledge relating to sustainable development. Understanding and knowledge of sustainability issues have been termed sustainability literacy, which is “… the understanding, skills, attitudes, and attributes to take informed action for the benefit of oneself and others, now and into a long term future” [12]. Older people living in different accommodation forms, ranging from independent-living alternatives to high-level care, may have different perceptions and knowledge of sustainability and sustainable development. According to Murray and Cotgrave [13], a sustainability literate person understands the need for change to a sustainable way of doing things individually and collectively, has sufficient knowledge and skills to decide to act in a way that favors sustainable development, and is able to recognize and reward other people's decisions and actions that favor sustainable development [14]. The first sustainability literacy surveys were originally developed to assess student sustainability knowledge, sustainable practices, attitudes concerning sustainability topics, and awareness of sustainability initiatives at the Arizona State University, Meredith College, and Maryland University in the USA.

Retirement villages are becoming accepted as a viable accommodation option and house over five percent of Australians aged 65 years and over. A survey of 76 older people living in not-for-profit retirement villages in South Australia found that they are both very conscious of resource consumption and concerned about energy costs [8]. To date, however, nothing is known of the detailed views of residents or of those living in for-profit retirement villages outside South Australia. Further north in Queensland, for example, the warmer climate and reputation for a more relaxed lifestyle are popular for retirees who want to relocate. Queensland has a reputation for having an entrepreneurial approach to business which provides an opportunity to incorporate attitudes of residents within the planning and development processes.

This research explores the perceptions of older people, living in a comparatively large and luxurious Queensland for-profit retirement village, regarding sustainable issues relating to environmental, economic, and social aspects of sustainability. This paper focuses on resident's sustainability concerns, impacts on activities of daily life, and sustainability literacy.

## 2. Methods

The study sought to investigate the sustainability literacy of residents living in a Queensland for-profit retirement village. Data were collected from October to December 2012. Ethical approval was granted by the Queensland University of Technology Human Research Ethics Committee. Approval from the target regiment village was also obtained to provide necessary assistance for data collection.

The survey was conducted in a private retirement village in a suburb of Brisbane, Queensland, Australia, located approximately 10 kilometers northwest of the Brisbane central business district (CBD) close to amenities such as transport, health services, stores, and libraries. The retirement village developer aspires to be a leader in sustainable property and infrastructure, focusing on transitioning to a low-carbon, energy-efficient, and resource-conservation model that reflects and advances social and environmental best practice. The village contains 254 one-, two-, or three-bedroom homes.

Based on sustainability literacy surveys for university students, a survey was developed focusing on attitudes and behaviors concerning sustainable development. The questionnaire included questions on environmental, economic, and social issues and comprised two main parts (see Table 1). The first part addressed older people's awareness of sustainability issues (using 1: strongly disagree, 2: disagree, 3: agree, and 4: strongly agree); and the second part explored the frequency of daily sustainability-related activities (using 1: never, 2: rarely, 3: usually, and 4: always). The unique aspects of older people living in retirement village were also taken into consideration in the questionnaire design.

Survey forms were placed in the village's community center's reception room from October 26, 2012, until December 4, 2012, with a covering letter explaining the purpose of the research.

## 3. Results

Sixty-five residents returned the completed questionnaire, providing an overall response rate of approximately 25%.

**Figure 1:**
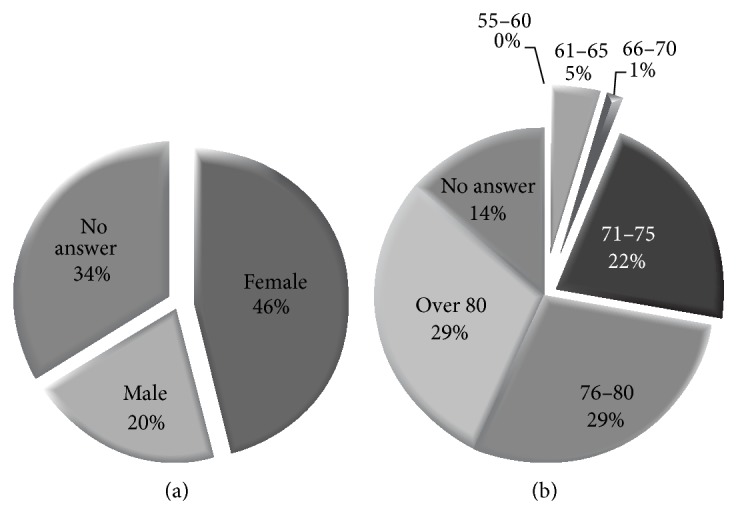
(a) Respondents' gender distribution. (b) Respondents' age distribution.

Figure 1 summarizes the respondents' demographics with the majority of the residents being female and older than 70 years.

The frequencies of degree of agreement of the importance of sustainability activities as well as the median score and interquartile ranges (IQR) are shown in Table 2. Most respondents agree or strongly agree on the importance of these activities to sustainable development. Most of the median scores of importance perceptions are 3 or higher, particularly for “recycling items when possible,” “buying local products,” and “cutting down on the use of products that are harmful to the environment,” where all the respondents agree on their importance. Interestingly, the majority (67%) of residents agree on the necessity of paying a higher price for a home that has environmentally friendly features.

Table 3 describes the frequencies of residents carrying out activities that relate to sustainability as well as the median score and interquartile ranges (IQR). More than 50% of respondents “always” separate landfill waste and recycled waste (63%) and turn off lights and electronic devices when not in use (52%). In contrast, 43% of respondents “rarely” create crafts from recycled materials or old stuff and 74% “never” use rainwater tanks for gardening or laundering.

**Figure 2:**
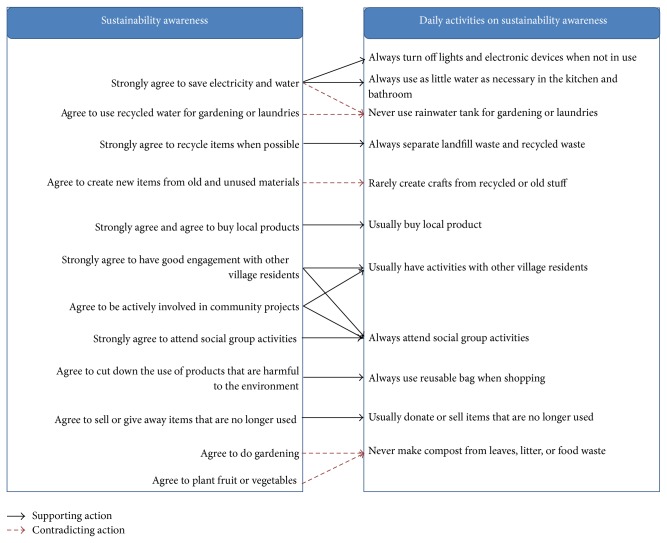
Relationship of sustainability awareness and daily activities.

Based on survey results, the relationship between sustainable awareness of respondents and their daily activities is drawn (see Figure 2). In general, high levels of sustainability awareness of respondents lead to high frequencies of sustainability activities in their daily life.

## 4. Discussion

Given their increasing number in our society, older people play an important role in fulfilling the sustainability agenda. It is predicted that a growing proportion of older people will live in retirement villages in the next 10 years. Understanding the attitude and behavior of retirement village residents is of great importance for the delivery of sustainable retirement villages in the future. The results of the questionnaire survey reveal that the majority of respondents have a sound understanding on the holistic aspects of sustainability, which requires the reconciliation of environmental and economic demands and the need for social equity. In other words, there was sufficient sustainable literacy within the senior citizen community living in the studied retirement village. In particular, the most important sustainability issue perceived by respondents was “reporting housing issues to the facility manager for repair.” This clearly indicated that respondents are fully aware of the effectiveness of resolving housing issues by having facility management professionals engaged. Similarly, respondents perceived the interaction with other village residents to be very important. This showed older people's attention to social sustainability-related issues. Environmental sustainability issues, such as energy, resource, and water efficiency, were also assigned high priority by respondents for achieving sustainability.

This sustainability literacy of retirement village residents is correlated with their behavior in terms of daily activities. A sustainable development not only protects the natural environment (environmentally sustainable) and increases economic growth (economically sustainable) but also promotes social progress that takes into account the needs of everyone (socially sustainable). For environmental sustainability, most respondents agree on the importance of energy and water saving, recycling, and using fewer products that are harmful to the environment. Furthermore, most respondents manage to save energy in their daily activities. For example, more than 90% of respondents use as little water as necessary and turn off lights and electrical devices when not in use. This confirms previous findings that older people in retirement villages are concerned about environmental issues and welcome more energy saving in their living environment [8, 15].

For economical sustainability, it is interesting to find that most respondents agreed with paying a higher price for homes with environmental friendly features. This is different from previous findings that although nearly all the residents in retirement villages would like to have their facilities more environmentally friendly, most are concerned about the costs involved and are reluctant to pay a higher price for an environmentally friendly home [8, 15]. The major reason for the difference is probably because the respondents in this previous research were residents in not-for-profit retirement villages, where affordability is of the highest importance to residents. The retirement village investigated in this research is a private one that has a very low ongoing vacancy rate (6–8%). This difference provides interesting implications for future retirement village planning. For example, the “baby boomer” generation represents the potential residents of retirement villages of the future. Considering that baby boomers normally have better financial circumstances than previous generations, they are likely to be more inclined to pay additional cost associated with sustainability features of living environment. The retirement village industry may want to take full consideration of this emerging demographical change and endeavor to satisfy the changing requirements involved.

For social sustainability, it is within expectations that most of the respondents agree with the importance of attending social group activities and having sufficient engagement with other village residents. In fact, almost all the residents have very good relationships with their neighbors and village managers and attend social group activities quite often (with 42% “usually” and 46% “always”). As confirmed by the findings of previous research, social activities, friendships, and social networks are important for the residents' quality of life [8, 16–18]. Older people need to remain socially active and it is crucial in personal functioning of later life [19, 20]. Retirement village developers should therefore create a living environment/community that facilitates the residents' social activities and social relationships that are critical for maintaining quality of life.

As shown in Figure 2, there is close connection between the respondents' sustainability awareness and frequency of daily sustainable activities. For example, as illustrated in Figure 2, if respondents “strongly agree” with saving electricity and water, they “always” turn off the lights and electrical devices when not in use and use as little water as necessary. However, although most respondents believe that it is important to recycle water, they never use rainwater tanks for gardening or laundering. This outcome is due to the lack of infrastructure to harvest rainwater and storm water within the village setting. Contrasts also exist between the “agree to gardening and planting fruit or vegetables” and “never” making compost for their gardens and between “agree to create new items from old and used materials” and “rarely create crafts from recycled or old stuff.” As the majority of residents in this village are over 75 years old and have comparatively reduced physical capabilities, the low frequency of conducting these sustainability activities does not necessarily imply a low level of sustainability literacy of respondents. Instead, this suggests an important implication for the retirement village industry in which the introduction of sustainability features should take full consideration of the physical limit of older people. Otherwise, as with many current communities, some public facilities will have physical barriers for older people [21]. Retirement villages should provide a supportive environment that increases accessibility to and usability of sustainability features.

## 5. Conclusions

The study described in the paper conducted a sustainability literacy survey of residents of a private retirement village in Queensland in order to explore their attitudes and activities relating to sustainability issues. The survey results show that most residents have sufficient literacy as they recognize the importance of environment protection and would like to lead a more environmentally friendly lifestyle. In addition, the majority of respondents are willing to pay a higher price for a living environment with sustainable features. Furthermore, almost all the respondents agree with the importance of social communications and good relationships with other residents in the village. Results also showed a close connection between village residents' sustainability literacy and their behavior in terms of daily activities.

These findings provide useful inputs into the type of sustainable features to incorporate into retirement villages. As residents are very aware of energy and water saving, energy-efficient designs (e.g., north-facing windows, open floor plan, and the location of internal walls) and materials (e.g., double-brick walls, double glazed windows, and thermally insulated roof) and water saving fixtures (e.g., in tap ware, toilets, and showerheads) can be adopted. In addition, given that retirement village residents regularly attend social group activities and recognize the importance of social sustainability in their quality of life, developers are recommended to incorporate socially friendly facilities into their villages, for example, by providing as a community center, game room and community garden, to facilitate social engagement and create a living environment that creates opportunities for residents to develop friendship networks and participate in a range of activities in the village and the wider community. Finally, considering that the majority of residents rarely exercise because of their physical limitations or lack of sustainability literacy, the incorporation of sustainable features in retirement villages should take into consideration their physical conditions and unique requirements. For example, a village bus (within and outside the village) would provide a welcome service. Furthermore, due to the changing demographic conditions of potential residents especially with impending retirement of the “baby boomer” generation, future retirement village planning should take full consideration of their unique requirements as they are likely to be wealthier, healthier, and more socially active than previous generations.

There are more than 1800 retirement villages in Australia and residents in villages of different types (not-for-profit or for-profit), different locations (urban or remote suburb) and different sizes, and so forth may have different perceptions and behaviors relating to sustainable development. For example, as this study has shown, issues concerning affordability can be quite different in different circumstances. Future research opportunities therefore exist to conduct a larger scale survey at national level in order to provide a full profile of the sustainable literacy of the residents of retirement villages.

## Figures and Tables

**(a):** 

Category	Sustainability awareness
Environment	Saving electricity and water
	Using recycled water for gardening or laundering
	Choosing more durable items to maximize their lifespan
	Cutting down on the use of products that are harmful to the environment
	Selling or giving away items that are no longer being used
	Creating new items from old and unused materials
	Recycling items when possible
	Reporting any housing issues to facility manager to repair

Economic	Paying a higher price for a home that has environmentally friendly features
	Buying local products
	Gardening
	Planting fruits or vegetables in own garden or community garden

Social	Attending social group activities
	Actively involved in community projects
	Having a good engagement with other village residents

**(b):** 

Category	Daily activities on sustainability awareness
Environment	Turning off lights and electronic devices when not in use
	Using as little water as necessary in the kitchen and bathroom
	Using a rainwater tank for gardening or laundering
	Using reusable bags when shopping
	Creating crafts from recycled material or old stuff
	Making compost from leaves, litter, or food waste
	Separating landfill waste and recycled waste

Economic	Donating or selling items that are no longer used
	Buying local products

Social	Attending social group activities, such as community meetings or community events, with other village residents
	Having activities such as community gardening, barbeque, sports, dancing, or making crafts with other village residents

**Table 2:** Percentage of respondents agreeing on the importance of sustainability activities.

Sustainability awareness	Degree of importance				Number	Median	IQR
	1	2	3	4			
Reporting housing issues to the facility manager for repair	0	2	32	**66**	65	4	1
Saving electricity and water	2	0	40	**58**	65	4	1
Recycling items when possible	0	0	46	**54**	65	4	1
Buying local products	0	0	50	**50**	64	3.5	1
Having a good engagement with other village residents	0	3	48	**49**	65	3	1
Attending social group activities	0	8	45	**47**	62	3	1
Paying a higher price for a home that has environmentally friendly features	3	17	**67**	13	60	3	0
Using recycled water for gardening or laundering	0	7	**64**	29	55	3	1
Being actively involved in community projects	0	13	**55**	32	62	3	1
Cutting down on the use of products that are harmful to the environment	0	0	**55**	45	64	3	1
Creating new items from old and unused items	2	29	**54**	15	61	3	1
Choosing more durable items to maximise their lifespan	0	2	**53**	45	64	3	1
Selling or giving away items that are no longer used	0	5	**50**	45	64	3	1
Gardening	8	18	**40**	34	62	3	1.75
Planting fruit or vegetables in own or community garden	3	31	33	**33**	58	3	2

Note: 1: strongly disagree, 2: disagree, 3: agree, and 4: strongly agree.

**Table 3:** Percentage of respondent carrying out sustainability activities.

Daily activities	Frequency				Number	Median	IQR
	1	2	3	4			
Separate landfill waste and recycled waste	13	6	17	**63**	63	4	1
Turn off lights and electronic devices when not in use	0	6	42	**52**	65	4	1
Use as little water as necessary in the kitchen and bathroom	0	3	48	**49**	65	3	1
Attend social group activities such as community meeting or community events with other village residents	3	9	42	**46**	65	3	1
Use reusable bags when shopping	5	19	33	**44**	64	3	1
Buy local products	2	3	**66**	30	64	3	1
Donate or sell items that are no longer used	0	6	**48**	45	64	3	1
Have activities such as community gardening, barbeque, sports, dancing, or making crafts with other village residents	6	17	**41**	36	64	3	1
Create crafts from recycled material or old stuff	37	**43**	16	5	63	2	1
Use rainwater tanks for gardening or laundering	**74**	16	5	5	62	1	1
Make compost from leaves, litter, or food waste	**39**	26	19	16	62	2	2

Note: 1: never, 2: rarely, 3: usually, and 4: always.

## References

[1] Brutland Report Our Common Future, Chapter 2: Towards Sustainable Development. World Commission on Environment and Development. http://www.un-documents.net/ocf-02.htm.

[2] Department of the Environment, Transport, the Regions (2000). *Building a Better Quality of Life: A Strategy for More Sustainable Construction*.

[3] Brandon P., Lombardi P. (2010). *Evaluating Sustainable Development in the Built Environment*.

[4] Guerra Santin O., Itard L., Visscher H. (2009). The effect of occupancy and building characteristics on energy use for space and water heating in Dutch residential stock. *Energy and Buildings*.

[5] Farag S., Lyons G. (2012). To use or not to use? An empirical study of pre-trip public transport information for business and leisure trips and comparison with car travel. *Transport Policy*.

[6] Pillemer K., Wagenet L. P. (2008). Taking action: Environmental volunteerism and civic engagement by older people. *Public Policy and Aging Report*.

[7] Bridge C. (2012). Cities, environmental stressors, ageing and chronic disease. *Australasian Journal on Ageing*.

[8] Barker J., Zuo J., Xia B., Zillante G. Sustainable retirement living: what matters.

[9] Pillemer K., Wells N. M., Wagenet L. P., Meador R. H., Parise J. T. (2011). Environmental sustainability in an aging society: a research agenda. *Journal of Aging and Health*.

[10] Gracia N., Moyle W., Oxlade D., Radford K. (2010). Addressing loneliness in a retirement village community: a pilot test of a print-delivered intervention. *Australasian Journal on Ageing*.

[11] Willis K., Scarpa R., Gilroy R., Hamza N. (2011). Renewable energy adoption in an ageing population: heterogeneity in preferences for micro-generation technology adoption. *Energy Policy*.

[12] Diamond S., Irwin B. (2013). Using e-learning for student sustainability literacy: framework and review. *International Journal of Sustainability in Higher Education*.

[13] Murray P. E., Cotgrave A. J. (2007). Sustainability literacy: the future paradigm for construction education?. *Structural Survey*.

[14] Parkin S., Johnson A., Buckland H., White E. (2004). *Learning and Skills for Sustainable Development: Developing a Sustainability Literate Society*.

[15] Zuo J., Xia B., Barker J., Skitmore M. (2014). Green buildings for greying people: a case study of a retirement village in Australia. *Facilities*.

[16] Buys L. R. (2000). Care and support assistance provided in retirement villages: expectations vs reality. *Australasian Journal on Ageing*.

[17] Croucher K. (2006). *Making the Case for Retirement Villages*.

[18] Xia B., Skitmore M., Zuo J., Buys L. (2014). Review of community facilities in Australian retirement villages: a content analysis. *Australasian Journal on Ageing*.

[19] Golden J., Conroy R. M., Lawlor B. A. (2009). Social support network structure in older people: underlying dimensions and association with psychological and physical health. *Psychology, Health and Medicine*.

[20] Grundy E., Read S. (2012). Social contacts and receipt of help among older people in england: are there benefits of having more children?. *The Journals of Gerontology B: Psychological Sciences and Social Sciences*.

[21] Fänge A., Iwarrson S., Persson Å. (2002). Accessibility to the public environment as perceived by teenagers with functional limitations in a south Swedish town centre. *Disability and Rehabilitation*.

